# Supplementary material for: Hyphopodium-Specific Signaling Is Required for Plant Infection by Verticillium dahliae
Source: J Fungi (Basel). 2023 Apr 18;9(4):484. doi: 10.3390/jof9040484 (PMC10143791; doi:10.3390/jof9040484)
Supplement: Supplementary file 1 [file jof-09-00484-s001.zip › jof-2319099-supplementary.pdf]

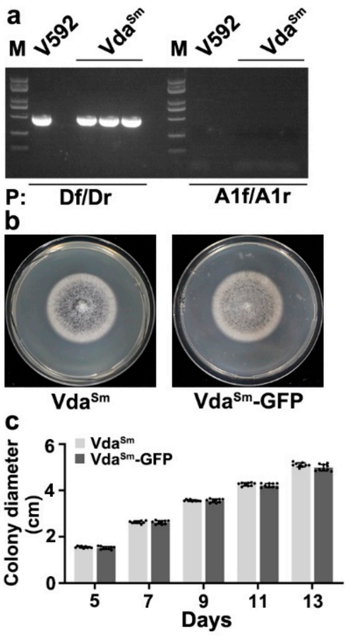

Figure S1. The colony morphology and growth rate of Vda^{Sm} and Vda^{Sm}-GFP on PDA plates. (a) PCR assays differentiated the *Verticillium* species. The primer pair Df/Dr amplified *V. dahliae* and V. *longisporum* strains, whereas A1f/A1r was specific to *V. longisporum*. V592 was used as a control. M indicated the marker; (b) The colony morphology of Vda^{Sm} and Vda^{Sm}-GFP on PDA plates; (c) The diameter of colonies at 5, 7, 9, 11 and 13 days. For each strain, 9 plates were repeated. No significant differences were noticed ($P > 0.05$, t-test). Error bar showed the standard deviations.

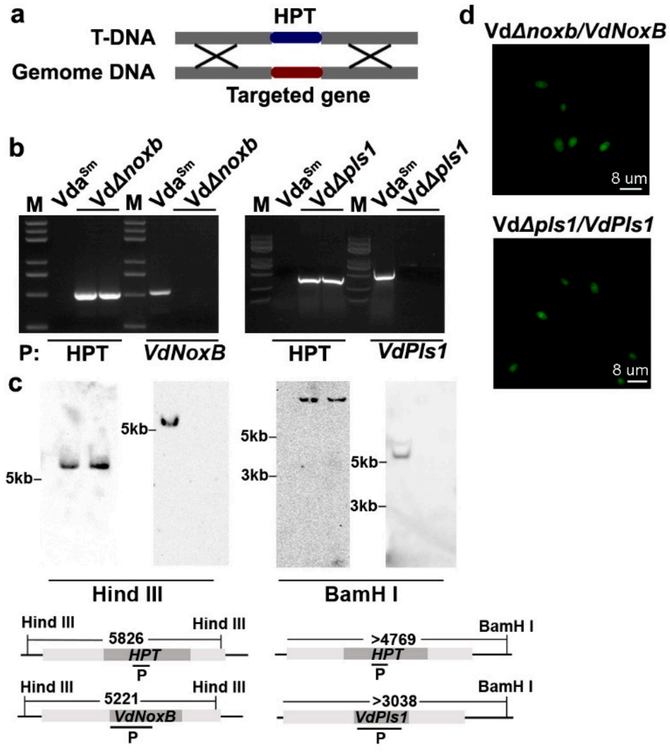

Figure S2. Construction of knockout mutants Vd*Δnoxb* and Vd*Δpls1*, and complemented strains Vd*Δnoxb*/*VdNoxB* and Vd*Δpls1*/*VdPls1*. (a) Schematic description of the homologous recombination event; (b) PCR analysis of targeted gene deletion mutants with special primers as indicated; (c) Southern blot identification of mutants with the *HPT*, *VdNoxB* and *VdPls1* probes as indicated. Genomic DNA was digested by Hind III and BamH I, respectively. The schematic diagrams showed the cleavage sites of

restriction enzymes and the expected fragment sizes; (d) Confocal micrograph of green fluorescence by Vd*Δnoxb*/*VdNoxB* and Vd*Δpls1*/*VdPls1*.

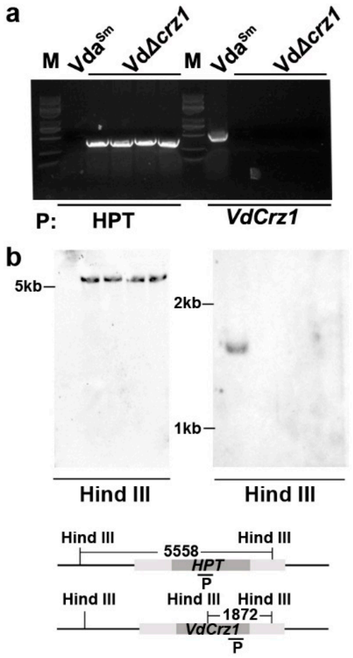

Figure S3. Identification of the *VdCrz1* knockout mutant. (a) PCR identification of the *VdCrz1* knockout mutant with the primers HPT and *VdCrz1* as indicated; (b) Southern blot analysis of *VdCrz1* gene deletion with *HPT* and *VdCrz1* probes as indicated. Genomic DNA was digested by Hind III. The schematic diagrams showed the cleavage sites of restriction enzymes and the expected fragment sizes.

Table S1. Primers were used in this study.

Name	Primer Sequence(5'-3')	Purpose
**ITS1**	TCCGTAGGTGAACCTGCGG	Sequencing
**ITS4**	TCCTCCGCTTATTGATATGC	Sequencing
**A1f**	AAGTGGAGCCCCGTATCTTGAAT	Sequencing
**A1r**	CAACTGGCAACAGGGCTTGAAT	Sequencing
**Df**	CCGGTCCATCAGTCTCTCTG	Sequencing
**Dr**	CTGTTGCCGCTTCACTCG	Sequencing
**KONoxB-U-F**	CTTGCTGAGGTCTTAATTAACAACAAGAGCCGCAATCAA	Cloning
**KONoxB-U-R**	AGTGCTGAGGCATTAATTAACGTGCTGGTAGTGCTATAATCGT	Cloning
**KONoxB-D-F**	CCCGCTGAGGACTTAATTAAAGGGGGCAAGGCGTGGAGCA	Cloning
**KONoxB-D-R**	CTCGCTGAGGGTTTAATTAAATGGAACACGCTCAAACACGTCG	Cloning
**KOPls1-U-F**	CTTGCTGAGGTCTTAATTAAAGCGCACTAGGTGCTTGCAAGT	Cloning
**KOPls1-U-R**	AGTGCTGAGGCATTAATTAAGTTGCTGGCGGTCTCGAGGACT	Cloning
**KOPls1-D-F**	CCCGCTGAGGACTTAATTAAACGACCGTAAAGCCGATCTC	Cloning
**KOPls1-D-R**	CTCGCTGAGGGTTTAATTAAGAGGCAAGGAAATGATGATCAGT	Cloning
**KOCrz1-U-F**	CTTGCTGAGGTCTTAATTAACTCCAGCGCTCATCTACCCT	Cloning
**KOCrz1-U-R**	AGTGCTGAGGCATTAATTAAGGTTGGGGACGTGTCGTGAGTA	Cloning
**KOCrz1-D-F**	CCCGCTGAGGACTTAATTAAGTGTGCCTTTGTTCTTCTTC	Cloning
**KOCrz1-D-R**	CTCGCTGAGGGTTTAATTAAAGAGGACCATGTTCCATAACG	Cloning
**pGKO-check-F**	GTCGACGGTATCGATAAGCTTGCTC	Checking
**pGKO-check-R**	CGGACATGCAGCTCACGCCTA	Checking
**HPT-F**	ATAAGAGTCACACTTCGAGCGCC	Checking
**HPT-R**	GCTCATGAGCGCTTGTTTCGG	Checking
**Pnoxb-NoxB-F**	TAAAACGACGGCCAGTGCCAAGCTTCTCCAGGTAACAGGGAAGA	Cloning
**Pnoxb-NoxB-R**	GCCCATCGAGTAGTAGTCCATCGTGCTGGTAGTGCTATAAT	Cloning

**NoxB-GFP-F**	AGCACGATGGACTACTACTCGATGGGCTC	Cloning
**NoxB-GFP-R**	AATTAAGCCGCCTCCGCCGAAGTTCTCCT TGCCCCATT	Cloning
**Ppls1-Pls1-F**	TAAAACGACGGCCAGTGCCAAGCTTGGGCACGG CACAAAGGGTCA	Cloning
**Ppls1-Pls1-R**	GATCTTGTTGACCATGTTGCTGGCGGTCTCGAGG ACTGT	Cloning
**Pls1-GFP-F**	GCCAGCAACATGGTCAACAAGATCCTCGCGACC TTCG	Cloning
**Pls1-GFP-R**	AATTAAGCCGCCTCCGCCGAGGCTGCGGTATCC GCT	Cloning
**pTef-Kar2-RFP-F**	AACCTCTAGAGGATCCATGTCGAGGTCTC GCAAC	Cloning
**pTef-Kar2-RFP-R**	CACCAGCACCGAATTCGAGCTCGTCGTGG GAGGC	Cloning
**HPT-probe-F**	CTTCTGCGGGCGATTTGTGTAC	Southern Blotting
**HPT-probe-R**	CATGTGTATCACTGGCAAACTGTG	Southern Blotting
**NoxB-probe-F**	GACTACTACTCGATGGGCTC	Southern Blotting
**NoxB-probe-R**	AATCTTCCTCAGGCGCACTCGT	Southern Blotting
**Pls1-probe-F**	GATGTGCTTTTCCTGATCAC	Southern Blotting
**Pls1-probe-R**	CGGTCCTCCAGTAGCACAGCAAG	Southern Blotting
**Crz1-probe-F**	ACGTTCCAGTGCAACCTGTGT	Southern Blotting
**Crz1-probe-R**	ATCAAACGAAGACCGTCCCGAAA	Southern Blotting
**NAT-probe-F**	TGGAGCTAGTGGAGGTCAAC	Southern Blotting
**NAT-probe-R**	GAGGCCCTTTCGTCTTCAAG	Southern Blotting
**β-tublin-qF**	AGCTCACCCAGCAGATGTTC	RT-qPCR
**β-tublin-qR**	TCGACCTCCTTCATGGCAAC	RT-qPCR
**VdCrz1-qF**	TTTACTCGCGCGTACAACCT	RT-qPCR
**VdCrz1-qR**	AACTTCTTCTCGCCCGAGTG	RT-qPCR
**VdLcc--qF**	TACCTCTTCCACTGCCACATC	RT-qPCR
**VdLcc-qR**	GTTTCCGCCAGCATTACCA	RT-qPCR
**VdRhom-qF**	CCCATGATTGGCCCCTCTTT	RT-qPCR
**VdRhom-qR**	CACCAAATGGGCTGTTGAGC	RT-qPCR
